# Crumbling crystals: on the dissolution mechanism of NaCl in water†

**DOI:** 10.1039/d4cp03115f

**Published:** 2024-10-11

**Authors:** Niamh O'Neill, Christoph Schran, Stephen J. Cox, Angelos Michaelides

**Affiliations:** a Yusuf Hamied Department of Chemistry, University of Cambridge Lensfield Road Cambridge CB2 1EW UK am452@cam.ac.uk; b Cavendish Laboratory, Department of Physics, University of Cambridge Cambridge CB3 0HE UK cs2121@cam.ac.uk; c Lennard-Jones Centre, University of Cambridge, Trinity Ln Cambridge CB2 1TN UK

## Abstract

Dissolution of ionic salts in water is ubiquitous, particularly for NaCl. However, an atomistic scale understanding of the process remains elusive. Simulations lend themselves conveniently to studying dissolution since they provide the spatio-temporal resolution that can be difficult to obtain experimentally. Nevertheless, the complexity of various inter- and intra-molecular interactions require careful treatment and long time scale simulations, both of which are typically hindered by computational expense. Here, we use advances in machine learning potential methodology to resolve at an *ab initio* level of theory the dissolution mechanism of NaCl in water. The picture that emerges is that of a steady ion-wise unwrapping of the crystal preceding its rapid disintegration, reminiscent of crumbling. The onset of crumbling can be explained by a strong increase in the ratio of the surface area to volume of the crystal. Overall, dissolution comprises a series of highly dynamical microscopic sub-processes, resulting in an inherently stochastic mechanism. These atomistic level insights contribute to the general understanding of dissolution mechanisms in other crystals, and the methodology is primed for more complex systems of recent interest such as water/salt interfaces under flow and salt crystals under confinement.

## Abstract 2

Machine-learned atomistic simulations reveal that NaCl dissolves *via* a crumbling mechanism.

## Graphical abstract

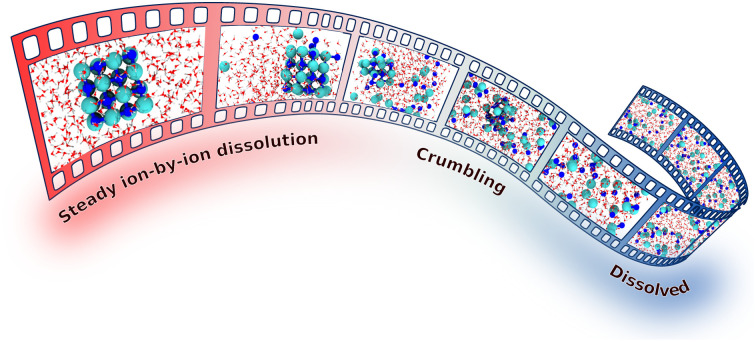

## 1 Introduction

Understanding the dissolution of crystals is vital for a myriad of pressing modern day challenges, from technological issues such as battery science^1^ and water desalination^2^ to drug bioavailability^3^ and geochemical weathering.^4^ The macroscopic process of dissolution is well described and understood from thermodynamics, where enthalpies of dissolution for example can be readily obtained from experimental techniques such as calorimetry.^5^ However despite well understood general theories describing the conceptually reverse process of nucleation,^6^ at a microscopic level our understanding of dissolution remains largely unresolved.

Dissolution is indeed an old problem,^7^ however with recent experimental developments, it is an exciting time for the field. Building on early atomic force microscopy studies of the water/NaCl interface by Xu *et al.*,^8^ sophisticated imaging techniques such as scanning tunneling microscopy^9^ and Bragg coherent diffraction imaging^10^ have given insight into low temperature surface ion dissolution events and the role of defects in the dissolution process. In addition, recent impressive high-resolution liquid cell transmission electron microscopy work has observed defect-mediated ripening of Cd–CdCl_2_ core–shell nanoparticles.^11^ The solid–liquid interface under flow has also been probed with a combination of surface-specific sum frequency generation spectroscopy and microfluidics experiments, revealing drastic changes to the equilibrium of dissolved ions.^12^ Recent single-molecule atomic-resolution real-time electron microscopy experiments have captured in real time a NaCl nucleus emerging during nucleation^13^ while *in situ* graphene liquid cell transmission electron microscopy has revealed the atomic mechanism of NaCl nucleation under confinement.^14^ However in general, obtaining atomic scale dynamical information from experiment remains a highly challenging endeavour.

In contrast, computer simulations conveniently grant access to the Ångstrom and femtosecond scale resolution required. A pertinent question for such studies relates to what is a good underlying model to describe the potential energy surface of ions in water? *Ab initio* methods, which aim to directly describe the electronic structure of a system have been used to resolve properties of solvated ions like their hydration structure,^15–21^ elucidating for example the differing effects of cations and anions on the local and global hydrogen bond network of water.^15^ Going beyond the solution phase, initial stages of dissolution have also been probed *via ab initio* molecular dynamics (AIMD) simulations,^22–25^ where for example the polarisability of ions is shown to be a key consideration.^25^

On the other hand, because of the long timescales and large system sizes required to resolve the dissolution process of even modestly sized nanocrystals, the vast majority of simulation studies up to now exploring dissolution itself have been performed using empirical force fields (see *e.g.* ref. 23, 26–34). Important insights have been obtained from these simulations that have significantly advanced the field, such as the fact that dissolution rates are highly controlled by the crystal structure, and that the existence of site specific barriers to dissolution implies a non-constant dissolution rate throughout the process.^35–39^ Nevertheless, computational studies have been limited to individual trajectories of dissolution events. However, a stochastic dissolution process is expected, as shown for example in larger scale studies in the geosciences.^40^ Therefore it is important to establish if the sequence of dissolving atoms can be rationalised at an atomistic level from an ensemble of dissolution trajectories. In addition, nucleation pathways are strongly dependent on solution concentration,^41–43^ raising the question whether dissolution follows the same predictable mechanism or if it is highly sensitive to the conditions.

In recent work, we have shown the importance of bridging the gap between *ab initio* approaches and classical force fields. For example, we have shown that the potential of mean force – a fundamental indicator of ion pairing – of a Na/Cl ion pair is highly sensitive to the underlying force field, with a significant spread in empirical force field predictions compared to *ab initio* methods such as density functional theory (DFT) and beyond.^44^ Fong *et al.* recently highlighted the inability of classical force field models to correctly describe the ion pairing behaviour of confined NaCl,^45^ while Panagiotopolous *et al.* have discussed the importance of an *ab initio* approach towards modeling dynamical properties of electrolytes.^46^

Therefore, to address the above questions regarding dissolution, there is a need for a computationally efficient exploration of multiple dissolution trajectories using a methodology that accurately describes the delicate changes in water–water and water–ion interactions that occur during dissolution. Fortunately, with the establishment of machine learning interatomic potentials (MLPs),^47–50^ as a routine tool in the field of computational materials science, accurate and efficient potentials can be developed for the treatment of complex processes such as dissolution. Such potentials yield the accuracy of *ab initio* methods but at a fraction of the computational cost. Of the many flavours of MLP methodologies and architectures, the approach reported in ref. 51 is particularly suitable as it enables the automated development and validation of MLPs capable of accurately treating complex aqueous systems with a suitably efficient evaluation time to access long trajectories.

Given the opportunities presented by the recent developments in MLP technology, we have performed a detailed study of NaCl dissolution. NaCl dissolution is a prototypical and widely studied system,^15,16,22,24,25,52–57^ because of its ubiquity and significance to phenomena including biological intracellular reactions^58^ and climate chemistry,^59,60^ not forgetting that two-thirds of the Earth is covered in salty water. We find that the dissolution process is highly dynamic. Many stochastic sub-processes combine to give an overall crumbling mechanism, in which a steady period of ion-wise dissolution precedes the rapid concerted disintegration of the crystal. This disintegration is governed by the steep increase of the surface to volume ratio of the crystal, corresponding to an unfavourable surface to bulk free energy that ultimately leads to the rapid collapse of the crystal.

## 2 The NaCl dissolution mechanism

**Fig. 1:**
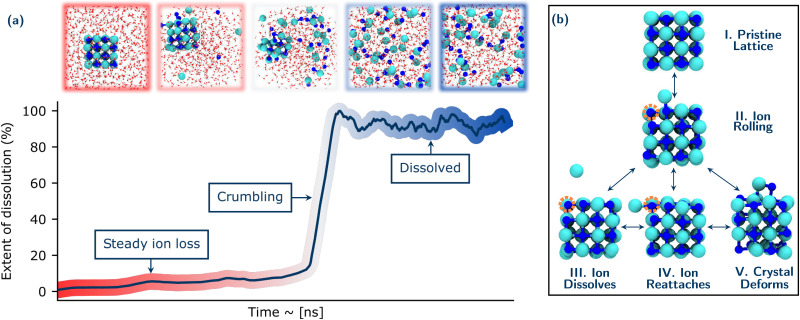
Overview of the NaCl dissolution process for a typical nanocrystal dissolution event. Panel (a) shows the extent of dissolution of a NaCl nanocrystal in water, which involves a steady loss of ions followed by a rapid crumbling event. Relevant snapshots along the trajectory are shown above the plot. Na^+^ and Cl^−^ ions are coloured in blue and cyan respectively and oxygen and hydrogen atoms are coloured in red and white, respectively. (b) Sub-processes occurring during the dissolution. The vacancies left by the ions moving are shown by the orange circles. For clarity in panel (b) the water molecules present in the simulation cell are not shown.

Fig. 1 provides an overview of a prototypical system considered and the NaCl dissolution process. Throughout this discussion, we show representative trajectories at one temperature but the phenomena observed hold for multiple trajectories at 330 K and 400 K. Further details of how our MLP was developed and extensively validated are provided in the ESI.† Full details of the systems and simulation protocols are given in the Methods section. However, in brief, we considered 64 and 216 ion NaCl nanocrystals in simulation boxes containing 1250 to 625 water molecules (giving NaCl concentrations when dissolved of 1.42, 2.84 and 5.61 mol kg^−1^). These concentrations were chosen to end up with different regimes from dilute to more concentrated solutions.

A typical dissolution trajectory is shown in Fig. 1(a), with the extent of dissolution of the crystal monitored over time. Relevant snapshots from the trajectory are shown in the upper panel. After a steady period corresponding to ions dissolving from typically low coordinated sites of the crystal into solution, there is a rapid increase in the rate of dissolution. This suggests the crystal reaches some critical point of instability after which it rapidly disintegrates or crumbles. Following this, the Cl^−^ and Na^+^ ions are fully solvated and the system has lost all crystalline order. These general observations for the dissolution mechanisms are consistent over all conditions studied and reproducible over the ensemble of trajectories at a given condition.

The early stages of dissolution up to the onset of crumbling comprise a set of dynamic processes occurring in equilibrium as shown in Fig. 1(b). The dissolution of any ion from the crystal is first preceded by the ion rolling onto the surface of the crystal (Structures I. & II.). This process involves a simultaneous reduction in coordination of the rolling ion with its neighbours in the lattice until it is just coordinated with one counter ion, and a corresponding increase in coordination of the ion with water. This behaviour is consistent with earlier AIMD studies on NaCl and Li_*x*_Mn_2_O_4_(001) surfaces.^25,61^ Moreover, there is a difference between the rolling behaviour of Na^+^ and Cl^−^: Na remains much more stable on the surface, while Cl^−^ is much quicker to dissolve into solution. This is in agreement with recent work by Silvestri *et al.*,^26^ in which they show that Na^+^ ions adsorb relatively strongly to the terrace sites when they move from the more stable kink sites, while Cl^−^ has much weaker minima on terrace sites. There are then several possibilities for the fate of this exposed ion: It can become further solvated by water and completely dissolve from the crystal (Structure III.) It can also roll back to its starting position and/or further roll to an orthogonal face (where it again has the possibility to dissolve from the crystal) (Structure IV.). The dissolved ion can also rejoin the crystal (at not necessarily the same location as it originally emerged). In these early stages before the rapid collapse of the crystal, other ions can also simultaneously roll and move about the crystal without necessarily any dissolution. This results in a deformation of the crystal shown in Structure V. The implications of these early dynamic processes with respect to the stochastic nature of dissolution will be discussed in detail in Section 3.

**Fig. 2:**
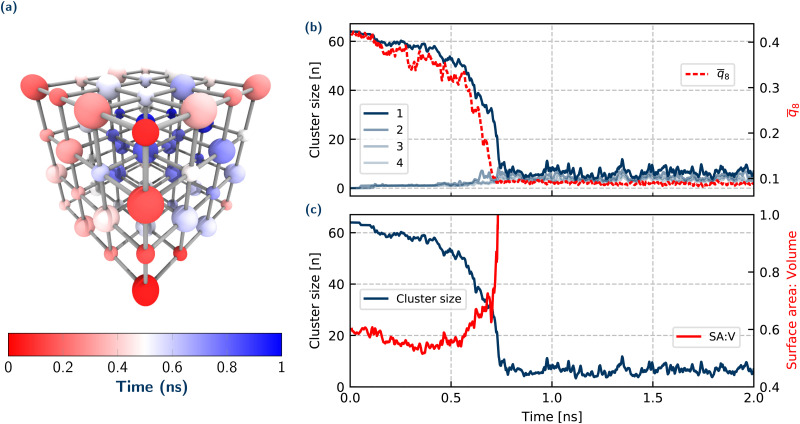
Summary of crystal and system properties for a dissolution trajectory at 2.42 mol kg^−1^. (a) Time taken for individual ions to dissolve in a single but representative NaCl nano-crystal, where the larger ions are Cl^−^ and smaller ions are Na^+^. Evolution over time of (b) cluster size of 4 largest ionic clusters and crystalline order parameter *q̄*_8_ (c) the ratio of surface area to volume (red, SA:V) of largest cluster (blue). A technical description of the *q̄*_8_ and surface area to volume order parameters are given in Section S.4 of the ESI.†

Fig. 2(a) shows a schematic of the typical ‘unwrapping’ of the crystal. The ions are coloured according to their time taken to dissolve for this specific crystal. Overall there is a preference for ions from low coordinated sites to dissolve first. We observe that dissolution is initiated at corner sites in the lattice, which has been previously noted in several papers.^22,25,52,56,62^ The dissolution of ions up to the point of crumbling proceeds in an approximately step-wise manner of ions of opposite charge (Cl^−^, Na^+^, Cl^−^…), thereby minimising charge accumulation on the crystal lattice, confirming earlier force-field simulations on the initial stages of dissolution.^56^ At each dissolution step (depending on its location) for an ion to dissolve it must break 3 (corner), 4 (edge), 5 (face) or 6 (centre) ionic bonds. Therefore the edge-wise unwrapping of the crystal shown in Fig. 2(a) follows the hypothesis that at each step, the number of ionic bonds broken is minimised. This holds true up to the point of rapid disintegration, where all atoms irrespective of their current coordination dissolve within a few picoseconds. We note that the asymmetry in the times to dissolve for equivalent lattice sites arises from the fact that only one trajectory is shown. Moreover, the stochastic fluctuations in the early stages of dissolution as described above mean that the nature of the initial water configuration surrounding the crystal will play a role in the times for specific ions to dissolve. By performing many averages over trajectories, the times to dissolve for equivalent lattice sites would eventually match the symmetry of the underlying crystal.

The crumbling observed in our simulations has been previously noted in classical force field simluations,^52,63^ where a decrease in lattice energy was hypothesised as the origin for the disintegration. However the mechanism of this process was not fully explored and we therefore set out to rationalise the origins of this crumbling by addressing a number of questions. Specifically: (i) does the crystal splinter into smaller crystallites or completely crumble into ions? (ii) Nucleation studies of NaCl in water, have shown that at very high concentrations there is an amorphous ionic structure before ions from solution form a nucleus^41,42^; is such an amorphous intermediate also relevant to dissolution? (iii) At what point does the crystal disintegrate, and is this crumbling event determined by the size of the remaining crystalline cluster or something else?

To answer these questions, we now analyse the nature of the crumbling mechanism, and attempt to quantify and describe the driving force for this event. Let us first address the question whether the remaining crystal splinters into smaller subclusters which is suggested in ref. 22. Another possibility is that it rapidly crumbles into individual ions. Monitoring the sizes of the 4 largest crystalline (ionic) clusters and the crystalline order of the largest cluster over time, as shown in Fig. 2(b) gives insight into the exact mechanism of the crystal collapse. Considering the sizes of the 4 largest ionic clusters, initially there is just one large cluster with 64 ions (*i.e.* the initial 4 × 4 × 4 crystal), which gradually decreases in size corresponding to the steady period of ions dissolving. At the point of crumbling, the number of ions in the largest cluster rapidly drops and immediately converges to a steady value of just one or two ions rapidly fluctuating over time (corresponding to short-lived ion pairs in solution). Since at no stage is there a second cluster with some intermediate number of ions, this implies that there is a complete disintegration of the crystal, rather than something resembling a shattering process.

To understand if an amorphous transition precedes crumbling, the crystalline order of the ions in the system has been monitored over time. *q̄*_8_ (defined in the ESI†) is a variation of the typical Steinhardt bond order parameter *q*_8_, and averages the bond order vectors over the first shell of neighbouring ions to provide a measure of ordering of the ions in the system. *q̄*_8_ (Fig. 2(b)) qualitatively tracks the evolution of the largest cluster size, whereby initially there is a steady decrease (but still within the range expected for a crystal) corresponding to the crystal decreasing in size. At a critical value of *q̄*_8_ ≈ 0.3, there is a sharp decrease in *q̄*_8_, corresponding to a rapid total loss of crystalline order in the system. Therefore for the (low) concentration regimes we explore, our results for dissolution reflect those in the nucleation literature, where there is a single step order to disorder transition, with no amorphous intermediate – although we note that in general, irrespective of concentration, there is no requirement for nucleation and dissolution mechanisms to be the same.

We have already suggested that the onset of crumbling occurs when the crystal shrinks to a certain (unstable) size. Across the simulated trajectories, there is a wide spread of crystal sizes at which the disintegration occurs (approximately 20–40 ions). So it is not size alone that governs the instability of the nanocrystal. To understand this behaviour we considered the surface area to volume ratio of the convex hull of the largest cluster. Panel (c) in Fig. 2 shows this surface area to volume ratio for the crystal over time up to the point of collapse. This gives a measure of the extent of interaction of the ions in the crystal with water over time. There is an initial decrease corresponding to a ‘rounding’ of the crystal, to a relatively constant value up to the point of crumbling. (This rounding is even more pronounced for the larger 6 × 6 × 6 crystal, Fig. S10, ESI†) At the onset of crumbling there is then a rapid increase in the surface area accessible to the water. This analysis highlights the importance of the solvent in the dissolution mechanism, where it plays a direct role in determining the crumbling. The volume term in this ratio can be understood in terms of the cohesive stabilising energy of the crystal phase, while the surface area term represents the energy penalty in forming an interface. The dominance of the surface area over the volume at the point of crumbling of the crystal lattice in this dissolution work highlights the delicate balance of inter-molecular interactions that ultimately determine the crystal stability. We note that obtaining a more quantitative metric for the free energy differences for the ions in the crystal or in solution could be addressed in future work utilising methods such as thermodynamic integration or metadynamics.^26^

In summary, this surface area to volume ratio is a simple parameter that explains the onset of disintegration of the crystal. It could also be used directly for other systems, thereby allowing for a simple and intuitive understanding of dissolution processes in general.

## 3 Stochastic nature of the NaCl dissolution process

Thus far, we have described the overall crumbling mechanism of NaCl dissolution in detail, which is completely general for multiple conditions of concentration and temperature. However a pertinent question relevant for many fields is whether the dissolution mechanism is deterministic. That is, given some initial conditions, can we predict at what crystal size/structure and after how long will the crystal dissolve? Previous *ab initio* studies^22,25^ and larger scale FF-based studies on dissolution have limited their focus to individual trajectories, thus restricting the conclusions that can be drawn regarding the mechanism in this respect. However in this work, access to multiple *ab initio* quality trajectories *via* machine learning based simulations has enabled this issue to be addressed through a thorough statistical analysis over multiple trajectories.

**Fig. 3:**
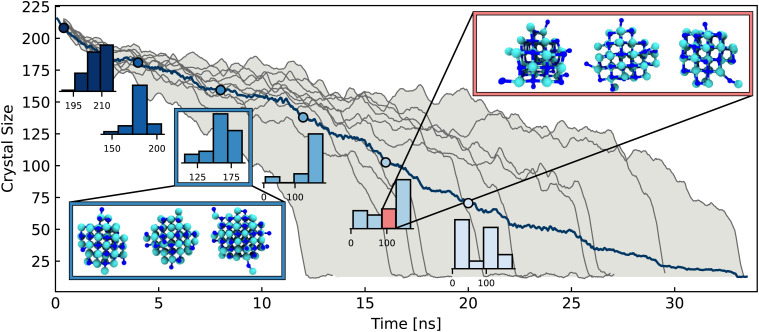
Overview of the stochastic nature of the NaCl dissolution process. The main panel shows the crystal size over time for 10 simulations at 330 K initialised with random velocities for a 6 × 6 × 6 nanocrystal. The range of fastest and slowest dissolving crystals is shaded in grey. The histograms show the distribution of crystal sizes across all trajectories for a given time window plotted along the mean crystal size (dark blue). Snapshots of crystals at 7 ns and with 87 ions are shown in the blue and red boxes respectively.

Fig. 3 shows the time evolution of the crystal size distribution for 10 trajectories of a 6 × 6 × 6 nanocrystal. The size distribution is initially very narrow at the beginning of the dissolution. However as dissolution proceeds along the 10 trajectories, the distribution widens significantly. After approximately 15 ns, there is a spread of over 100 ions in the crystal sizes along different trajectories. While all trajectories follow the overall crumbling mechanism described in Section 2, this broadening of the distributions illustrates the diverse microscopic paths taken by the crystals *en route* to dissolution. We previously discussed in Section 2 the highly dynamic equilibrium of microscopic subprocesses such as ions rolling on the crystal surface and lattice rearrangement occurring during the steady period of ion dissolution from the crystal. These are rooted in inherent stochastic system fluctuations such as lattice and molecular vibrations (on the order of fs) and water dipole and hydrogen bond reorientations (on the order of ps). Therefore the wide variation in the crystal structures at a given time (despite all simulations starting from the same pristine crystal structure) can be directly attributed to the stochastic nature of these microscopic sub-processes. Given the relatively small sizes of the systems studied here, one would expect a much larger spread of times in macroscopic systems. In addition to the range of crystal sizes for different trajectories at a given time, for a given crystal size, there are also numerous possible structures, as shown in the inset of Fig. 3, with 3 representative examples of a crystal with 87 atoms.

These diverse structures again arise from a combination of the stochastic microscopic subprocesses. The diversity in microscopic mechanism can also be observed for the other concentration conditions studied here. The largest 6 × 6 × 6 crystal (5.61 mol kg^−1^) takes much longer to dissolve and has a much broader range of dissolution times than the 4 × 4 × 4 crystals (1.42 mol kg^−1^ and 2.84 mol kg^−1^), as shown in Fig. S10 (ESI†). This broad spread of times can be attributed to an increase in probability of ions rejoining the 6 × 6 × 6 crystal, since it is surrounded by the lowest volume of water, but also there is a cumulative effect of each possible stochastic sub-process for every dissolution step. While a distribution of dissolution times is to be expected, a 2 or 3-fold spread in times is particularly noteworthy. In summary, the dissolution of NaCl therefore proceeds *via* an overall crumbling mechanism, within which, there is rich structural variety of the dissolving crystal owing to the inherently stochastic microscopic system fluctuations.

## 4 Conclusion

Despite dissolution being one of the most ubiquitous processes on Earth, major gaps in understanding at the atomistic level persist. In this work we provide a first-principles description of the mechanism of NaCl dissolution, through the application of an automated framework to develop accurate MLPs. We have generated multiple trajectories (>300 ns in total) with *ab initio* equivalent accuracy, under a range of conditions of NaCl dissolving. We have established a general crumbling mechanism of NaCl dissolution, where following a steady period of ion-by-ion loss, the crystal reaches a critical point of instability and collapses in a concerted fashion. The sequence of dissolving ions in the steady period of dissolution is such that the number of ionic bonds broken and net charge on the crystalline lattice is minimised. The overall stability of a crystalline cluster in water can be straightforwardly reasoned by a delicate balance of interactions between the ions and water, measured *via* the surface area to volume ratio of the crystal. This simple and physically intuitive concept could be tested on other ionic crystals in water as well as non-ionic systems such as molecular crystals. The crumbling mechanistic insights we have provided are primed for experimental measurement, including confinement controlled monitoring of the dissolution and Bragg coherant diffraction imaging, as have already been successfully applied to crystalline systems.^12,13^

We have shown that the overall nature of the dissolution mechanism is highly stochastic, comprising a dynamic equilibrium of sub-processes such as ions rolling, dissolving, rejoining and deformations of the lattice in the crystal. These arise from the stochastic hydrogen bond formation/breaking and thermal vibrations in the crystal and influence the relative stability of the crystal, and therefore the overall time taken for dissolution.

Studies of dissolution of crystalline and non-crystalline materials in the earth sciences have observed a broad variation in the rate constant of dissolution of up to 2–3 orders of magnitude.^40,64^ which they attributed to the inhomogeneity of the initial crystal surface. Given our observations of a stochastic dissolution mechanism in the nanoscopic regime, exploring this feature of the mechanism *via* multiscale simulation methods such as kinetic Monte Carlo would be a very interesting next step. Moreover, leveraging the ever-increasing computational efficiency in MLP implementations, especially with the rise of GPU-based high-performance computing, sets the stage for improved statistics regarding processes on the nanometric scale.

Our observations regarding the dynamic nature of the crystal are also very amenable to further experimental studies. They suggest a large variation in dissolution mechanism under flow compared to confinement. In the former case the ions are rapidly removed from the surface - similar to the lower ion concentration conditions we have shown here, while confinement essentially traps the ions, creating a high concentration of ions in the vicinity of the surface. We propose that revisiting the microfluidics^12^ and electron microscopy^13^ experiments described in the Introduction, with a focus on different concentration conditions would be highly insightful.

The accurate treatment of the electronic structure of the system required to capture relevant interactions, yet at a computational cost that facilitates the simulation of many dissolution trajectories is crucial for the observations made here. The high transferability of the methodology and validation process is amenable to the study of other ionic salt systems. Indeed, having now understood NaCl dissolution – in terms of a minimization of charge and of bonds broken, a balance of ion-solvent interactions determining crystal stability, and the dynamic nature of the dissolution process – our work sets the stage for a generalised theory of dissolution of ionic crystals.

For example, the recent emergence of so-called ‘universal’ machine learning potentials,^65^ now allows for the efficient development of models for a range of crystal compositions. Computing the solubility of the ML model is also an important next step, given the large differences from experiment in solubilities predicted by different force field models.^66^ Typical approaches involving large-scale coexistance simulations^27,67,68^ would still be largely beyond the capabilities of the ML models, however thermodynamic integration approaches to obtain crystal and solution free energies would be a possible route.^66^ Finally, this methodology and the insights we have now obtained are primed for application to more challenging and complex systems of recent interest, including highly concentrated electrolyte solutions^69,70^ and electrolyte solutions under confinement.^71^

## 5 Methods

### 5.1 Machine learning potential

Machine learning potentials provide a direct functional relationship between atomic positions and forces/potential energy. This bypasses the otherwise computationally expensive requirement to solve Schrödinger-like equations and systems of much larger length and timescales than feasible with AIMD based approaches are now accessible. Building upon the seminal work of Behler and Parrinello in neural network based ML potentials,^72^ the recent development of an active learning approach using a committee of NNPs has enabled the systematic development and validation of NNP representations of the potential energy surface for complex aqueous systems^51,73^

Full details on the model are given in the ESI,† however briefly, the model comprised a committee of 8 Behler-Parinello NNPs comprising identical architecture of 2 hidden layers with 25 neurons in each hidden layer. Our model was iteratively trained on forces and energies from *ab initio* molecular dynamics simulations, on computationally accessible systems containing relevant interactions such as bulk water, solvated ion pair *etc* (for full details see ESI†) in the generalised gradient approximation using the rev-PBE functional with Grimme's D3 dispersion correction,^74^ while using a Coulomb baseline to incorporate the correct long-range electrostatics, evaluated with particle mesh Ewald summation. This baseline uses TIP3P model parameters^75^ for water and point charges of +/−1 for Na and Cl. This setup has been shown to be accurate for aqueous systems, and further details on functional selection and long-range electrostatics are described in the ESI.†

The model was iteratively improved over multiple generations such that the relevant configuration space was accurately covered in the overall training set with an energy and force root mean square error (RMSE) of 1.3 meV per atom and 38.0 meV Å^−1^, respectively. The total training set comprised 2127 configurations (energies) and 1 386 832 forces, yielding a robust model which was extensively validated in a series of tests incorporating static and dynamical properties of selected test systems. Full details are given in the ESI,† but the approach taken was to compare energies, forces, structural information such as radial distribution functions and vibrational densities of states to data obtained from AIMD. This is a standard approach of validating MLPs and ensures that the potential energy surface (PES) of the MLP faithfully represents the underlying *ab initio* PES for the relevant configuration space. Finally, having access to on-the-fly committee uncertainties along a trajectory builds confidence that the results reported are in the interpolation reigime of the model. Moreover, the same uncertainty can be used if the model is applied to other situations to identify failures in the model and where additional training is required.

### 5.2 MD simulations

The systems used in all dissolution simulations described comprised a 4 × 4 × 4 (6 × 6 × 6) NaCl nanocrystal with 32 (108) Na/Cl atoms using a computed lattice constant of 5.72 Å.^76^ The 4 × 4 × 4 nanocrystal was surrounded by 625 (1250) water molecules in a cubic simulation cell of side length 27.8 Å (34.1 Å), giving dissolved NaCl concentrations in water of 2.84 mol kg^−1^ and 1.43 mol kg^−1^ respectively. The 6 × 6 × 6 crystal was surrounded by 1068 waters in a cubic simulation cell of side length 34.1 Å giving a dissolved NaCl concentration in water of 5.61 mol kg^−1^.

We note that the cluster at the point of crumbling here is not equivalent to a critical nucleus in CNT. For the concentration regimes explored here, the critical nucleus would be expected to be much larger.^41^ To test the robustness of our proposed crumbling mechanism, we performed simulations on the two lowest concentration systems (2.84 mol kg^−1^ and 1.43 mol kg^−1^) using the classical Joung Cheatham force field for ion–ion interactions with the SPC/E water model.^66^ This has a computed solubility of 3.7 mol kg^−1^, and therefore we can be sure that our systems are below saturation when fully dissolved for this model. Full details and results are given in the ESI,† however we observe qualitatively the same crumbling mechanism, with similar cluster sizes at the point of crumbling. Therefore despite not having the solubility of the ML model, we can be confident that this mechanism is not simply a reverse of CNT. Moreover we do not observe any correlation between the disintegration threshold for the crystal and the overall NaCl concentration, where the main factor influencing the disintegration is instead the surface area to volume ratio.

MLP simulations were all carried out using the CP2K/Quickstep code in the NVT ensemble and at a constant temperature of 330 K (chosen to be consistent with previous literature^77^) maintained using the CSVR thermostat.^78^ At each concentration, 10 trajectories were simulated using the same initial configuration and initialised with random velocities drawn from the Boltzmann distribution at the target temperature of 330 K. Simulations were run for over 15 ns (5.61 mol kg^−1^), 2 ns (2.84 mol kg^−1^) and 1 ns (1.42 mol kg^−1^) until the crystal was fully dissolved. The same simulation procedure was carried out for 10 trajectories of the 2.42 mol kg^−1^ concentration at 400 K for further validation of the generalisability of our conclusions. These results are included in the ESI.† Overall over 300 ns of *ab initio* quality machine learning based simulations were performed, far beyond the capabilities of AIMD simulations. For a representative trajectory (20 ns, ∼3500 atoms), this corresponds to roughly 7600 node hours (AMD EPYC 7742, 2.25 GHz, 128 cores per node, 256 GB memory per node).

Further details for computing parameters including cluster size, *q̄*_8_ and surface area to volume ratio of the crystal are given in the ESI.†

## Code availability

All simulations were performed with publicly available simulation software (n2p2, CP2K), while the active learning package is available at GitHub (https://github.com/MarsalekGroup/aml).

## Data availability

All data required to reproduce the findings of this study is available at GitHub (https://github.com/niamhon/nacl-dissolution/.)

## Conflicts of interest

There are no conflicts to declare.

## Supplementary Material

CP-026-D4CP03115F-s001
