# Supplementary material for: Crumbling crystals: on the dissolution mechanism of NaCl in water
Source: Phys Chem Chem Phys. 2024 Oct 11;26(42):26933–42. doi: 10.1039/d4cp03115f (PMC11483817; doi:10.1039/d4cp03115f)
Supplement: CP-026-D4CP03115F-s001 [file CP-026-D4CP03115F-s001.pdf]

Supplementary Information (SI) for Physical Chemistry Chemical Physics.
This journal is © the Owner Societies 2024

Supporting Information for: Crumbling Crystals: On the Dissolution Mechanism of NaCl in Water

Niamh O'Neill,[1,2,3] Christoph Schran,[2,3,a)] Stephen J. Cox,[1,3] and Angelos Michaelides[1,3,b)]

[1)] *Yusuf Hamied Department of Chemistry, University of Cambridge, Lensfield Road, Cambridge, CB2 1EW, UK*

[2)] *Cavendish Laboratory, Department of Physics, University of Cambridge, Cambridge, CB3 0HE, UK*

[3)] *Lennard-Jones Centre, University of Cambridge, Trinity Ln, Cambridge, CB2 1TN, UK*

[a)] Electronic mail: cs2121@cam.ac.uk

[b)] Electronic mail: am452@cam.ac.uk

CONTENTS

S1. Selecting DFT functional S2

S2. Development of Machine Learning Potential S5

Automated Work Flow S5

Details of Model S6

Validation S9

$2 \times 2 \times 2$ NaCl nanocrystal S9

Validation of Dissolution Process S10

Long-range Effects S12

S3. Molecular dynamics simulations S14

System setups S14

Simulation details S15

Force field pre-equilibration details S16

AIMD simulation details S17

S4. Analysis Details S17

Steinhardt order parameters S17

Coordination Numbers and clusters S18

S5. Additional Results S20

MLP S20

FF S20

References S21

S1. SELECTING DFT FUNCTIONAL

Two modern DFT functionals that have been shown to perform well for liquid water simulations[S1,S2] - the dispersion corrected GGA functional revPBE-D3[S3,S4] and the strongly constrained and appropriately normed (SCAN) meta-GGA functional[S5]- were selected for an initial benchmark study to determine the most suitable XC functional for the *ab initio* simulations upon which the MLP would be trained. It should be noted that dispersion

corrected hybrid functionals have been shown to provide slightly better results for liquid water simulations;[S6,S7] however initial simulations with revPBE0-D3 showed there is approximately a six-fold increase in computational cost, rendering longer time-scale AIMD simulations unfeasible for the envisaged system sizes.

Na/Cl ion in water

To study the performance of SCAN and revPBE-D3 for ion-water simulations, four separate AIMD simulations were performed for both revPBE-D3 and SCAN for Na^+ in water and Cl^- in water. The Na(Cl)-O RDF g(r) are compared to experimental values in Figure S1 and Table I. Both SCAN and revPBE-D3 are in good agreement with the position of the first experimental Cl-O peak (R^1_{max}), where both are within 0.07 Å of the experimental data. However the height $g(R^1_{max})$ is underestimated by both by approximately 15%. SCAN and revPBE-D3 both qualitatively agree with the position of the second peak maximum, but they both tend to overstructure compared to experiment. The experimental Na-O peak is more accurately reproduced by SCAN than revPBE-D3. The revPBE-D3 peak is shifted 0.17 Å from the experimental value, and the peak height is 27% lower. This is also in agreement with previous studies, where SCAN has been shown to outperform GGA functionals in reproducing the sodium-water solvation structure.[S8] Despite a lack of experimental data for the second solvation shell, revPBE-D3 is in much closer qualitative agreement with SCAN than for the first peak.

TABLE I. Summary of structural properties of solvated sodium/chloride ions obtained from RDFs g(r) computed from AIMD simulations with SCAN and revPBE-D3 at 300 K. Experimental reference data for Cl-O and Na-O were obtained from neutron diffraction[S9] and X-Ray diffraction[S2] data respectively. Difference from experiment is shown in brackets.

	Cl-O			Na-O		
	SCAN	revPBE-D3	Experiment	SCAN	revPBE-D3	Experiment
R^1_{max} (Å)	3.10 (0.07)	3.19 (0.02)	3.17	2.36 (0.00)	2.53 (0.17)	2.36
$g(R^1_{max})$	2.83 (0.62)	2.70 (0.51)	3.21	5.79 (0.08)	4.30 (0.43)	5.87

The O-O RDF for both solvated sodium and chloride ion systems was also computed and compared to experiment in order to evaluate the performance of the two functionals for water. This analysis is shown below in Figure S2. In both cases, SCAN significantly overstructures water compared to revPBE-D3 and experiment, with a first peak maximum

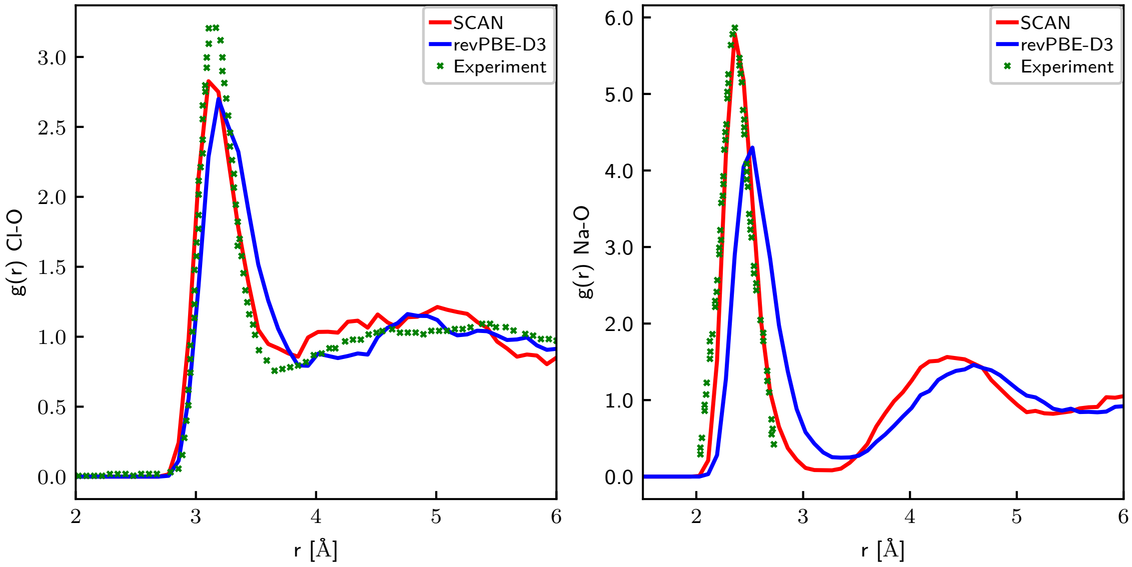

FIG. S1. Radial distribution function g(r) for Cl-O (left) and Na-O (right) comparing revPBE-D3 and SCAN with experiment. Cl-O experimental RDF obtained from neutron diffraction data[S9] and Na-O experimental RDF obtained from X-Ray diffraction data.[S8]

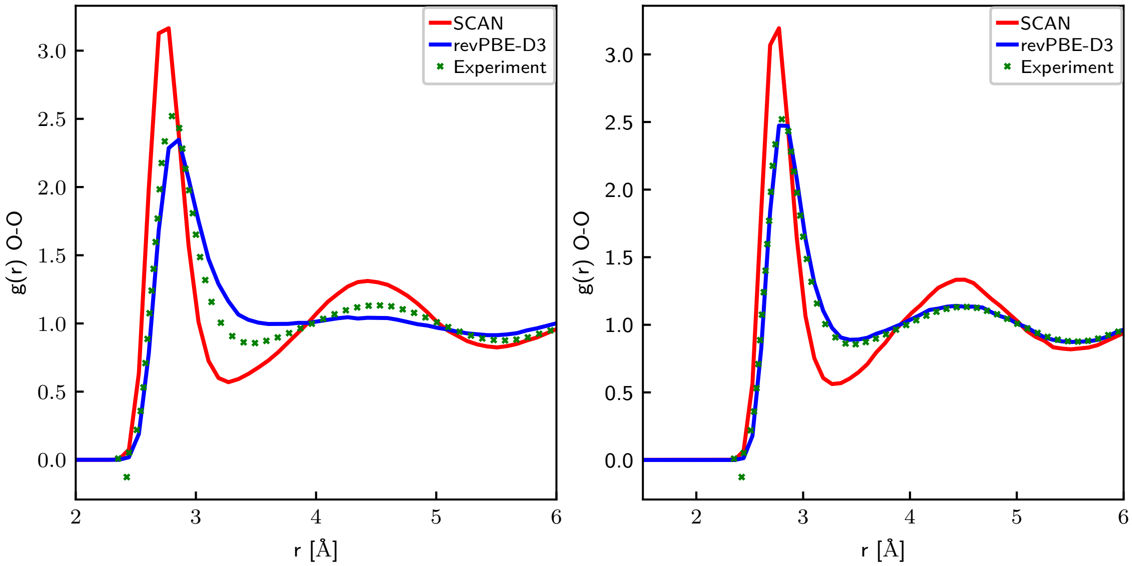

FIG. S2. Radial distribution function g(r) for O-O for Cl^-/H_2O (left) and Na^+/H_2O (right). Compared to experimental data taken from Ref. S10.

approximately 25% greater than experiment. This is consistant with literature results, in which Duignan et al. compared the performance of revPBE-D3 and SCAN in simulations of liquid water at 300 K.[S8] revPBE-D3 reproduced well the experimental O-O radial distribution function (RDF), with the first peak maximium and magnitude coinciding to within 0.1 Å and 2% respectively, while SCAN overstructured water, with a first peak RDF maxi-

mum approximately 20% larger than experiment. Galib et al. also report a computed water density with revPBE-D3 of 0.962 ± 0.029 gcm^{-3} within 4% of the experimental density of 0.997 gcm^{-3},[S2] compared to a computed density by Wiktor et al. of 1.05 gcm^{-3}.[S11]

In summary, the decision between SCAN and revPBE-D3 is not completely clear-cut and both functionals have advantages and disadvantages. revPBE-D3 performs better for liquid water than SCAN. With respect to the solvated ions, revPBE-D3 is on par with SCAN for the Cl-O interactions, however performs significantly poorer for Na-O. Nevertheless, revPBE-D3 reproduces experimental data much better than SCAN with respect to the O-O RDF. As a footnote, SCAN is also two-thirds more expensive than revPBE-D3. Therefore, the AIMD trajectories generated using revPBE-D3 were chosen for the initial development of the C-NNP training set and revPBE-D3 was used for generation of subsequent AIMD data and during the active learning stages of the MLP development.

S2. DEVELOPMENT OF MACHINE LEARNING POTENTIAL

Automated Work Flow

The procedure for developing the committee neural network potential (C-NNP) was followed as described in Ref S12. The general approach taken in developing the C-NNP was to treat different relevant systems and conditions individually with active learning, generating a training set for each condition and then combining the individual training sets to generate the final model. (We note that there are many active learning strategies that can lead in the end to converged models as for example in the ANIx model)[S13]

The model was iteratively improved over five generations of such active learning rounds, with each generation targeting a specific region of phase space. The overall development over these five generations of the model is summarised in Figure S3. An initial potential was trained on forces and energies obtained from *ab initio* simulations described in Section S3 comprising bulk water, individual Na^+/ Cl^- ions in water, an Na^+/ Cl^- ion pair in water and a small 2×2 nanocrystal in water. This model was then used under different temperatures to generate structures along the dissolution trajectory of the prototypical 4x4x4 nanocrystal (Generation 2). Later generations of the model targeted solution and intermediate structures along the dissolution trajectory (Generations 3+4) and highly concentrated solutions

(Generation 5). The development of the model used the Python AML active learning package described in Ref. S14. For a given active learning iteration, 20 random structures from a reference trajectory were used to initialise the model. After training 8 NNP members, forces and energies of 2000 randomly selected structures from the reference trajectory were predicted to ascertain the force and energy committee disagreements. 20 structures with the largest mean force disagreement were added to the training set for the next round of active learning. Convergence was reached when new structures added to the training set did not improve the committee disagreement between points already in the training set, indicating the training set was sufficiently diverse. The training set of the final C-NNP model contained 2127 structures. The overall force and energy training root mean square error (RMSE) for the model are 38.0 meV/Å and 1.3 meV/atom respectively. Given the complexity of the system under study, this compares well to a RMSE of 70 meV/Å quoted in Ref. S15 for a simpler system of large scale MD simulations of water using NNPs trained on DFT.

Details of Model

The chemical environment around each atom was described using a general set of atom-centered symmetry functions.[S16] There are 10 radial and 4 angular functions for each pair and triplet of atoms, following Ref. S12. All symmetry functions used a cutoff function of angular cosine form with a cutoff radius of 12 Bohr. The committee was comprised of 8 NNP members, of identical architecture with 2 hidden layers and 25 neurons in each layer. In all cases, random sub-sampling was performed to introduce variability between the committee members, where 10% of the total set of structures were discarded. The weights and biases of the NNPs were optimised using the n2p2 code.[S17] Individual models during active learning were optimised for 15 epochs, while the final C-NNP model used in simulations was optimised for 50 epochs.

To ensure that long-range effects are accurately captured by the machine learning potential, we explicitly incorporated long-range effects beyond the cutoff of the symmetry functions (12 Bohr). The predicted energy can in general be written as a sum of short range and long range contributions (E_{sr} and E_{coul} respectively): $E_{\text{tot}} = E_{\text{sr}} + E_{\text{coul}}$. The long-range model was thus trained on the difference between the standard short-ranged model and the Coulomb contribution, calculated using point charges of +/- 1 respectively for Na

and Cl and using TIP3P model parameters for water.[S18] We used this model in all production simulations, where the Coulomb contributions were explicitly included via particle mesh ewald summation. Details on the validation of the final model and the role of long range interactions in these simulations are presented in the next Section.

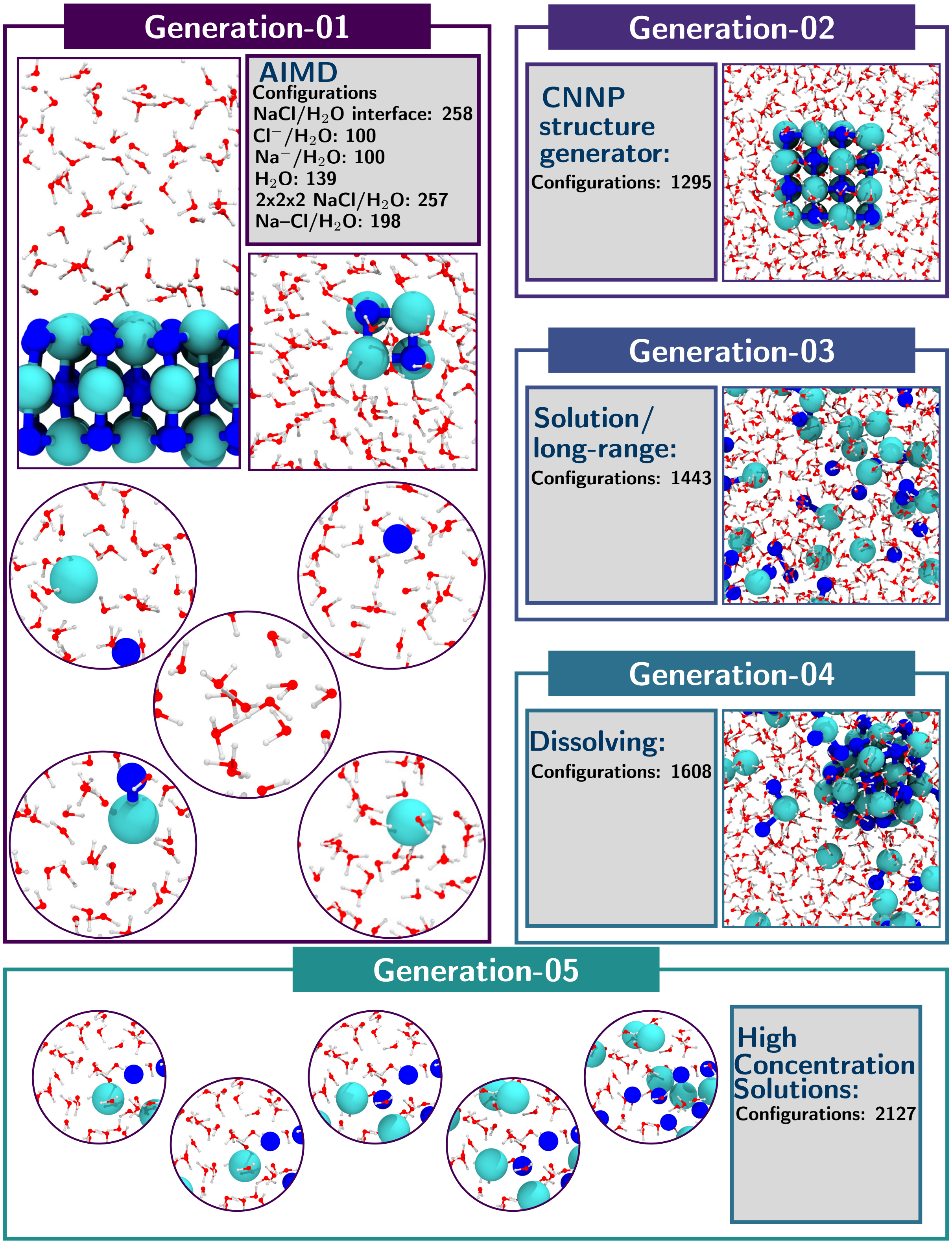

FIG. S3. Summary of iterative development of MLP over 5 generations. The number of configurations included from each AIMD simulation is given in Generation-01. Thereafter, the number of configurations is the total number of structures in the training set for the given generation.

Validation

The system studied is complex, so care was taken to set up a series of validation tests that incorporates relevant subsystems and properties.

$2 \times 2 \times 2$ *NaCl nanocrystal*

The static and dynamical properties of the largest accessible NaCl nanocrystal in water for AIMD simulations (2×2×2 NaCl surrounded by 229 waters - 695 atoms) was analysed using a benchmarking scheme proposed in Ref. S12, comparing AIMD to the C-NNP predictions and shown in Figure S4. The radial distribution functions (RDFs) $g(r)$ for all the atom species of the system gives a good assessment of the ability of the C-NNP to predict thermodynamic properties. The vibrational density of states (VDOS) for each element gives information on the vibrational modes of the system, and thus the dynamics of inter- and intra-atomic interactions. The forces on the atoms are sensitive to the local environment, and give additional insight via which the quality of the ML model can be evaluated. RDF and VDOS predictions are all in essentially perfect agreement with the AIMD reference trajectory while the force predictions and the DFT reference are very well correlated. It should be noted that the slight discrepancy in the VDOS between the C-NNP and AIMD at higher frequencies is likely due to the large plane wave cutoff required in the DFT calculations to obtain converged Na forces (Discussed in further detail in Section S3). Nevertheless the symmetry between Na and Cl VDOS spectra is reassuring.

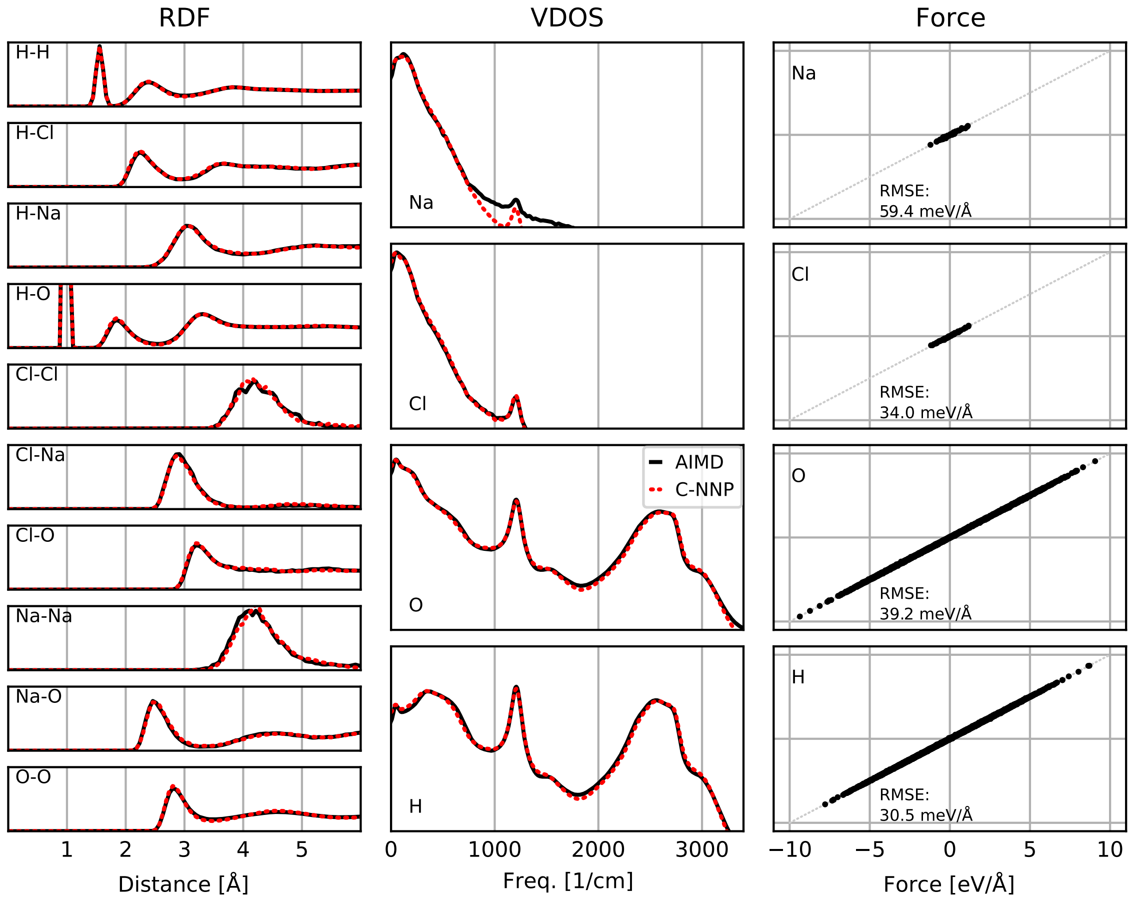

FIG. S4. Benchmark tests of long-range C-NNP model predictions of structural and dynamical properties of a $2 \times 2 \times 2$ NaCl nanocrystal in water as well as force prediction. The comparison between AIMD and the C-NNP RDFs, VDOS and force correlation are shown in the left, middle and right panels respectively.

Validation of Dissolution Process

As the most rigorous of tests for the C-NNP we validate the ML model for the actual dissolution process of a 4x4x4 nanocrystal in solution by comparing the force and energy predictions of the model and the corresponding DFT forces and energies for a set of structures uniformly selected along the dissolution process. It is particularly valuable since the model has not been explicitly trained on these structures. First, an extensive MD simulation with the developed model was performed in order to sample the dissolution process of the 4x4x4 nanocrystal in solution. A variety of structures were selected to capture all of the relevant configurations along the trajectory; from the intact NaCl crystal lattice, to the initial stages of dissolution to the end point of the fully solvated ions in solution. Next, the DFT reference

energies and forces of these 50 structures were computed. The resulting correlation of the forces is shown in Figure S5. The RMSE values for both forces (37.0 meV/Å) and energies (0.3 meV/atom) compare very favourably to similar reactive systems, which have been studied using machine learning potentials, such as the work by Behler et al. in Ref. S19 who quote a force and energy RMSE for a model describing proton transport at ZnO/H_2O interfaces of 140.4 meV/Å and 1.0 meV/atom respectively.

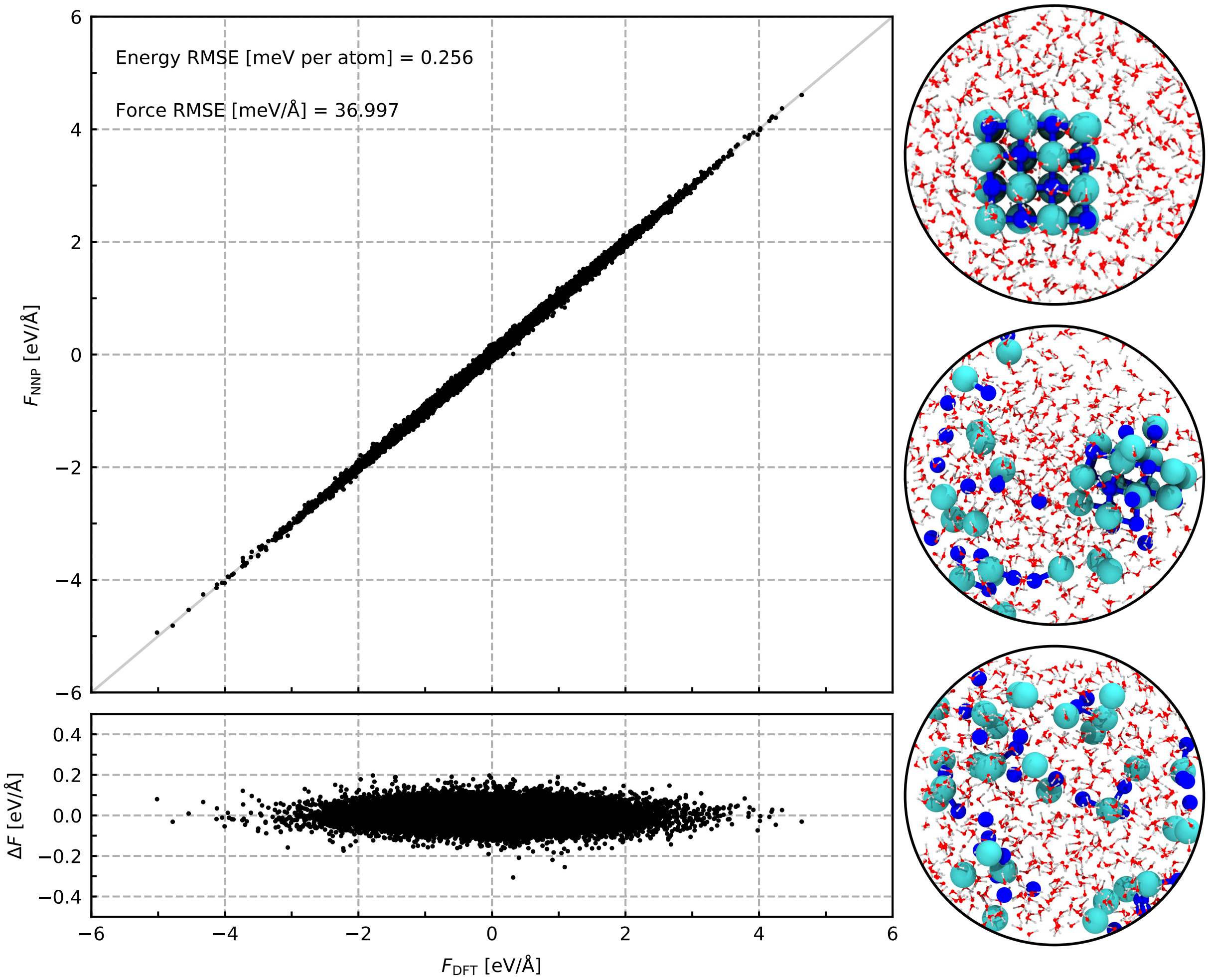

FIG. S5. Correlation plot for C-NNP predicted forces and corresponding reference DFT forces, with light grey line showing a perfect correlation coefficient of 1. Representative snapshots of the variety of structures tested are shown on the right hand side of the figure.

Long-range Effects

In order to evaluate the effect of explicitly incorporating long range effects into the model structural properties (RDFs) of the 2.48 mol/kg NaCl solution were computed. These are compared to the short range model in Figure S6. Both predictions are in very good agreement for all cases of water, ions and ion-water RDFs. Nevertheless small discrepancies between long- and short-range model predictions in the cases of the Cl-Cl and Na-Cl RDFs prompt us to use the long-range model for production runs, given the additional physical information included in the model. In general however this analysis suggests that overall for our simulated systems, long-range effects do not have a substantial impact. This is in agreement with observations by Yue et al. using a similar approach of including a Coulomb baseline[S20] of .

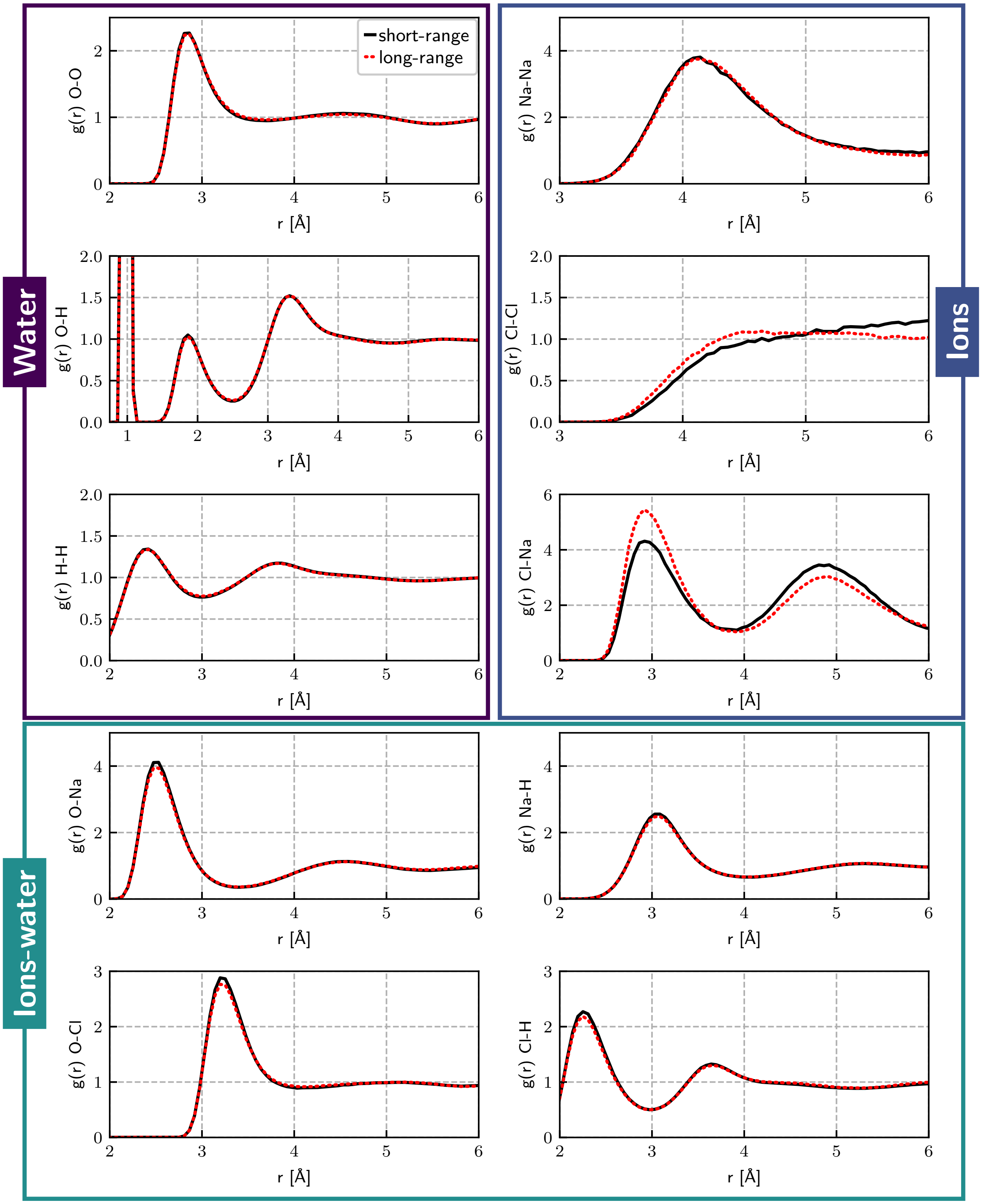

FIG. S6. Comparison of long and short-range models in predicting NaCl solution properties. RDFs for 2.48 mol/kg NaCl solution for both long- and short-range model predictions are shown in red and black respectively.

In summary this set of validation tests confirm the overall quality and accuracy of the C-NNP model. The model has performed comparably to or exceeded literature values on a variety of systems and properties that are very relevant for the dissolution process, from small scale studies of single ions in solution to systems sizes and interactions that will be explored in the detailed analysis of the dissolution process.

S3. MOLECULAR DYNAMICS SIMULATIONS

System setups

All simulations were carried out in an orthorhombic simulation cell with periodic boundary conditions in x, y, z directions. Details of the simulation setups for all systems are shown in Table II.

TABLE II: Details of systems simulated. L is (unless stated) the cubic box dimensions in Å.

**System**	**Simulation Details**	**Snapshot**
Bulk H_2O	$N_{water} = 64$<br>L = 12.42 Å	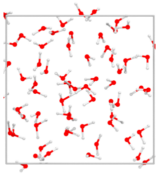
Na^+ / H_2O	$N_{water} = 63$<br>$N_{Na} = 1$<br>L = 12.42 Å	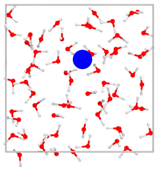
Cl^- / H_2O	$N_{water} = 63$<br>$N_{Cl} = 1$<br>L = 12.42 Å	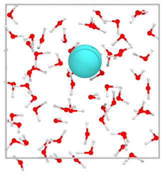
Na^+–Cl^- contact ion pair	$N_{water} = 62$<br>$N_{Na} = 1$<br>$N_{Cl} = 1$<br>L = 12.42 Å	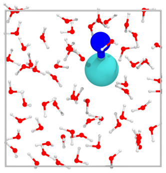
Na^+–Cl^- solvent-separated ion pair	$N_{water} = 62$<br>$N_{Na} = 1$<br>$N_{Cl} = 1$<br>L = 12.42 Å	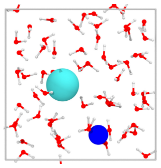

NaCl / H_2O interface	N_{water} = 89<br>N_{Na} = 36<br>N_{Cl} = 36<br>L(x,y,z) = (16.2, 12.1 58.7) Å	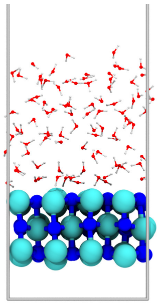
2 × 2 × 2 NaCl nanocrystal	N_{water} = 229<br>N_{Na} = 4<br>N_{Cl} = 4<br>L = 19.8 Å	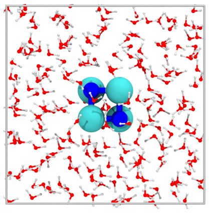
4 × 4 × 4 NaCl nanocrystal (2.48 mol/kg)	N_{water} = 625<br>N_{Na} = 32<br>N_{Cl} = 32<br>L = 27.8 Å	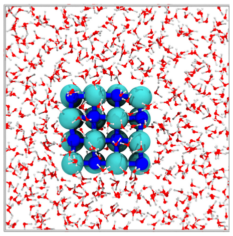
4 × 4 × 4 NaCl nanocrystal (1.42 mol/kg)	N_{water} = 1250<br>N_{Na} = 32<br>N_{Cl} = 32<br>L = 34.1 Å	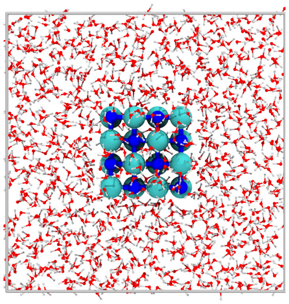
6 × 6 × 6 NaCl nanocrystal (5.61 mol/kg)	N_{water} = 1068<br>N_{Na} = 108<br>N_{Cl} = 108<br>L = 34.1 Å	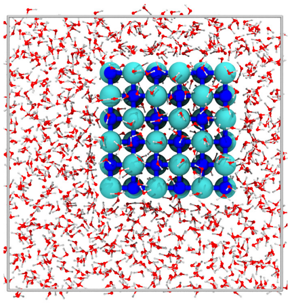

Simulation details

All MD/AIMD simulations were performed using the CP2K/Quickstep code.[S21] AIMD simulations were used to generate reference data for development of the model as shown in Table III, with pre-equilibrated structures obtained from force field simulation. C-NNP

TABLE III. Details of simulation lengths performed with AIMD.

**AIMD**	
**System**	$\mathbf{T_{sim}}$
Bulk H2O	100 ps
Na / H2O	80 ps
Cl / H2O	70 ps
NaCl/H2O interface	70 ps
Na–Cl contact ion pair	36 ps
Na–Cl solvent-separated ion pair	36 ps
2 x 2 x 2 NaCl nanocrystal	25 ps

TABLE IV. Details of simulation lengths for production runs performed with C-NNP.

**C-NNP Production**	
**System**	$\mathbf{T_{sim}}$
$4 \times 4 \times 4$ NaCl nanocrystal (2.84 mol/kg)	$10 \times >2$ ns
$4 \times 4 \times 4$ NaCl nanocrystal (1.42 mol/kg)	$10 \times >2$ ns
$6 \times 6 \times 6$ NaCl nanocrystal (5.61 mol/kg)	10×10 - 30 ns

production simulations as shown in Table IV were initially equillibrated with fixed ions using the C-NNP model.

Force field pre-equilibration details

A 1 ns force-field based MD simulation was first performed on all Na/Cl containing systems from Table III to obtain a pre-equilibrated initial configuration for subsequent AIMD simulations. The Born-Mayer-Huggins potential for NaCl[S22] and TIP3P model for water[S18] were used and the water/NaCl interactions were described by the parameters given by Lynden-Bell et al. in Ref. S23.

Additional production simulations as described in Section S5 were performed using the Joung Cheatham (JC) model for ion-ion interactions, with SPC/E water model and ion-water interactions described by LJ crossed interactions using the Lorentz-Berthelot combination rules. These parameters were taken from Ref. S24.

AIMD simulation details

Prior to all AIMD simulations, a short constrained AIMD simulation, keeping ions fixed, was performed on all the systems given in Table III containing ions, to obtain a well-equillibrated water structure around the Na and Cl ions. These were used as starting configurations for simulations in NVT ensemble within the generalised gradient approximation using the revPBE functional[S4] with Grimmes' dispersion correction.[S25] The electronic density was partitioned into core and valence contributions, with core electrons described using the norm-conserving Goedecker, Teter and Hutter (GTH) pseudopotentials.[S26] Na 2s and 2p electrons were also treated explicitly given the well-known issue of non-linear core-valence exchange/correlation. Valence electrons were described using the MOLOPT TZV2P basis set.[S27] Stochastic errors for Na forces due to insufficient basis set convergence were observed in additional DFT convergence tests for the cutoff of the plane-wave representation of the charge density. Therefore a plane wave cutoff of 1200 Ry was used to obtain accurate forces. Deuterium masses were used in lieu of hydrogen, allowing a 1 fs timestep and reducing errors due to neglecting nuclear quantum effects through classical propagation of the nuclei. Simulations were performed at 300 K, maintained using the CSVR thermostat..[S28] All AIMD simulations were at least 40 ps long (except for $2 \times 2 \times 2$ NaCl nanocrystal which was 25 ps due to computational cost.) and can be found in Table III.

S4. ANALYSIS DETAILS

Steinhardt order parameters

The Steinhardt order parameter q8 is a particular case of a larger set of bond order parameters based on spherical harmonics.[S29] To distinguish between solid and liquid-like ions we first compute the complex vector $q_8(i)$ for each particle i, where $N_b(i)$ is the number of nearest neighbours of particle i, and m runs from -8 to +8.

$$q_{8,m}(i) = \frac{1}{N_b(i)} \sum_{j}^{N_b(i)} Y_{8,m}(\mathbf{r}_{i,j}) \tag{S1}$$

$Y_{8,m}$ are the spherical harmonics and $\mathbf{r}_{i,j}$ connects particles i and j. Following Lechner et al.[S30] we then compute an averaged form of this local bond order parameter by averaging the local $q_{8,m}(i)$ vectors over the particle i and its surroundings:

$$\bar{q}_8(i) = \sqrt{\frac{4\pi}{2l+1} \sum_{m=-l}^{l} |q_{8,m}(i)|^2} \tag{S2}$$

where

$$\bar{q}_{8,m}(i) = \frac{1}{\tilde{N}_b(i)} \sum_{k=0}^{\tilde{N}_b(i)} Y_{8,m}(k) \tag{S3}$$

and $\tilde{N_b}(i)$ are the neighbours of particle i

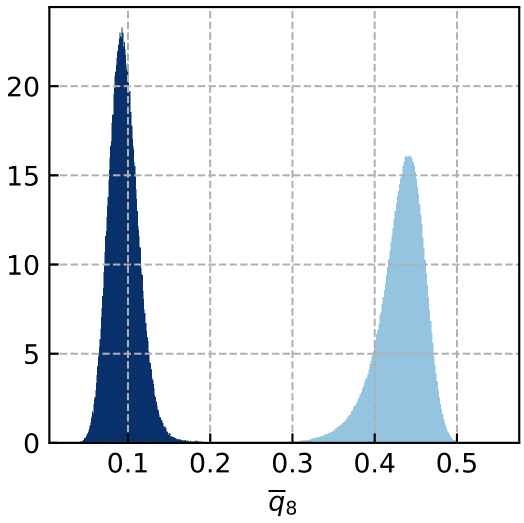

FIG. S7. $\bar{q}_8$ distributions for ideal cases of pristine NaCl lattcice (light blue) and solvated Na/Cl ions (dark blue).

Computation of these bond order parameters was performed using the Freud[S31] python package.

Coordination Numbers and clusters

Computation of all coordination numbers and clusters were performed using Plumed,[S32] and analysis carried out using Python. The CN of an atom i with a set of atoms in A is

defined by:

$$\mathrm{CN}_i = \sum_{j \in A} s_{ij} \tag{S4}$$

where s_{ij} is a smooth switching function with a range of [0,1]:

$$s_{ij} = \left(\frac{1 - \left(\frac{r_{ij}}{r_0}\right)^6}{1 - \left(\frac{r_{ij}}{r_0}\right)^{12}} \right) \tag{S5}$$

r_{ij} is the interatomic distance between atom i and atom j. r_0 for Na-O (2.80 Å) and Cl-H (2.60 Å) were obtained from literature values,[S33] while r_0 for Na-Cl was taken such that the CNs of the pristine lattice coincided with the integral up to the first minimum of the Na-Cl RDF (3.5 Å). An ion was defined as dissolved if it has a Na-O/Cl-H CN less than 1 for more than 8 ps. We show that this definition is not sensitive to changes in the time chosen in Figure S8. The cluster size distributions were computed by detecting coordinated ions

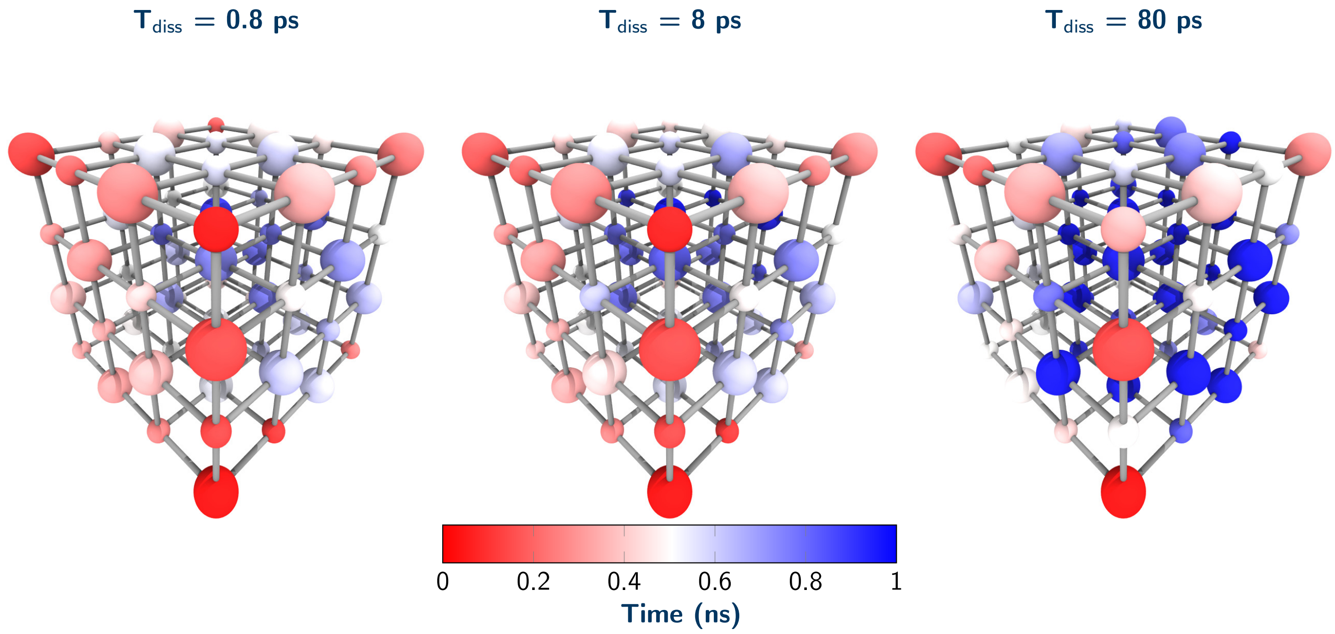

FIG. S8. Sensitivity of dissolution behaviour of ions based on definition of time spent with CN<1.

using a threshold of a ion-ion CN > 2, and then using these atoms as input for a depth first search graph reduction algorithm to identify the sets of ions clustered together.

To compute the surface area to volume ratio of the dissolving crystal, a DFS algorithm was implemented using the Freud Python package[S31] in order to identify the ions in the

largest cluster at a given time. The convex hull of this set of points was then obtained (provided there were > 3 ions in the cluster), and the surface area to volume ratio of the hull was computed.

S5. ADDITIONAL RESULTS

MLP

Results from identical simulations as described above for the C-NNP at 2.48 mol/kg NaCl concentration at 400 K are shown in Figure S9. The additional results for the 1.42 mol/kg and 2.48 mol/kg trajectories at 330 K are also shown in Figure S9.

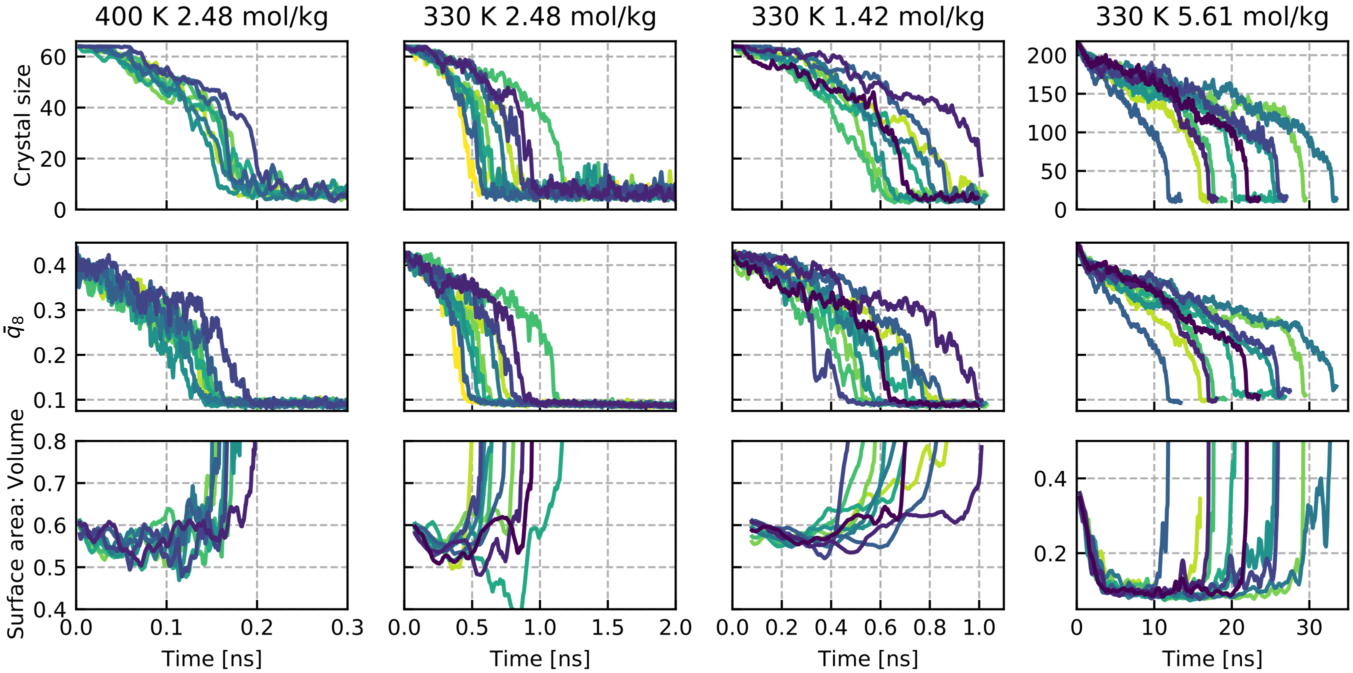

FIG. S9. Crystal size, $\bar{q}_8$ and surface area:volume ratio for ten simulated trajectories at 400 K (2.48 mol/kg) and 330 K (2.48 mol/kg, 1.42 mol/kg and 5.61 mol/kg).

FF

Results from identical simulations as described above for the C-NNP at 1.42 mol/kg and 2.48 mol/kg at 330 K are shown in Figure S10 for a JC/SCP/E force-field model.

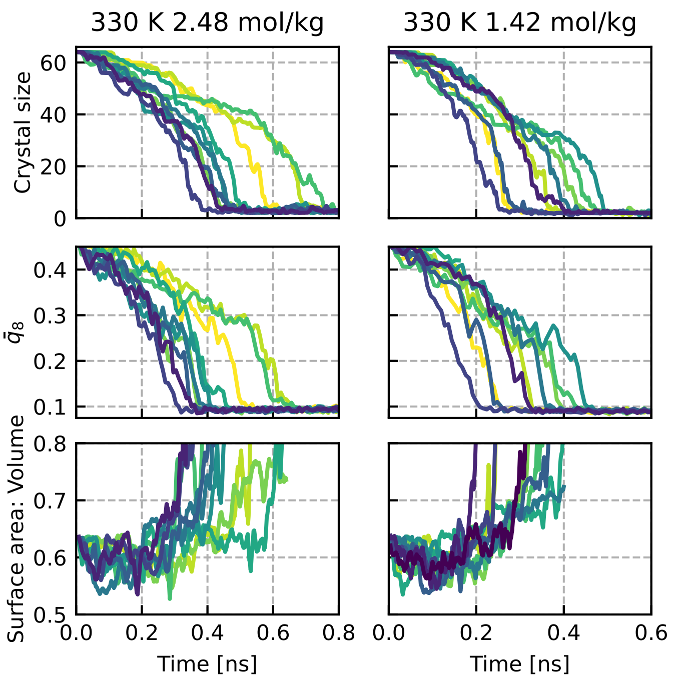

FIG. S10. Crystal size, $\bar{q}_8$ and surface area:volume ratio for ten simulated trajectories with JC/SPC/E at 330 K (2.48 mol/kg and 1.42 mol/kg).

REFERENCES

[S1] L. Zheng, M. Chen, Z. Sun, H. Y. Ko, B. Santra, P. Dhuvad, and X. Wu, "Structural, electronic, and dynamical properties of liquid water by ab initio molecular dynamics based on SCAN functional within the canonical ensemble," J. Chem. Phys. **148**, 164505 (2018).

[S2] M. Galib, T. T. Duignan, Y. Misteli, M. D. Baer, G. K. Schenter, J. Hutter, and C. J. Mundy, "Mass density fluctuations in quantum and classical descriptions of liquid water," J. Chem. Phys. **146**, 244501 (2017).

[S3] J. P. Perdew, K. Burke, and M. Ernzerhof, "Generalized Gradient Approximation Made Simple," Phys. Rev. Lett. **77**, 3865–3868 (1996).

[S4] Y. Zhang and W. Yang, "Comment on "Generalized Gradient Approximation Made Simple"," Phys. Rev. Lett. **80**, 890 (1998).

[S5] J. Sun, A. Ruzsinszky, and J. Perdew, "Strongly Constrained and Appropriately Normed Semilocal Density Functional," Phys. Rev. Lett. **115**, 036402 (2015).

[S6] O. Marsalek and T. E. Markland, "Quantum Dynamics and Spectroscopy of Ab Initio Liquid Water: The Interplay of Nuclear and Electronic Quantum Effects," J. Phys. Chem. Lett. **8**, 1545–1551 (2017).

[S7] A. P. Gaiduk, J. Gustafson, F. Gygi, and G. Galli, "First-Principles Simulations of Liquid Water Using a Dielectric-Dependent Hybrid Functional," J. Phys. Chem. Lett. **9**, 3068–3073 (2018).

[S8] T. T. Duignan, G. K. Schenter, J. L. Fulton, T. Huthwelker, M. Balasubramanian, M. Galib, M. D. Baer, J. Wilhelm, J. Hutter, M. Del Ben, X. S. Zhao, and C. J. Mundy, "Quantifying the hydration structure of sodium and potassium ions: Taking additional steps on Jacob's Ladder," Phys. Chem. Chem. Phys. **22**, 10641–10652 (2020).

[S9] A. K. Soper and K. Weckström, "Ion solvation and water structure in potassium halide aqueous solutions," Biophys. Chem. **124**, 180–191 (2006).

[S10] L. B. Skinner, C. Huang, D. Schlesinger, L. G. Pettersson, A. Nilsson, and C. J. Benmore, "Benchmark oxygen-oxygen pair-distribution function of ambient water from x-ray diffraction measurements with a wide Q-range," J. Chem. Phys. **138**, 074506 (2013).

[S11] J. Wiktor, F. Ambrosio, and A. Pasquarello, "Note: Assessment of the SCAN+rVV10 functional for the structure of liquid water," J. Chem. Phys. **147**, 164505 (2017).

[S12] C. Schran, F. L. Thiemann, P. Rowe, E. A. Müller, O. Marsalek, and A. Michaelides, "Machine learning potentials for complex aqueous systems made simple," Proc. Natl. Acad. Sci. U. S. A. **118** (2021).

[S13] J. S. Smith, R. Zubatyuk, B. Nebgen, N. Lubbers, K. Barros, A. E. Roitberg, O. Isayev, and S. Tretiak, "The ani-1ccx and ani-1x data sets, coupled-cluster and density functional theory properties for molecules," Scientific Data 2020 7:1 **7**, 1–10 (2020).

[S14] C. Schran, K. Brezina, and O. Marsalek, "Committee neural network potentials control generalization errors and enable active learning," J. Chem. Phys. **153**, 104105 (2020).

[S15] T. Morawietz, A. Singraber, C. Dellago, and J. Behler, "How van der Waals interactions determine the unique properties of water," Proc. Natl. Acad. Sci. **113**, 8368–8373 (2016).

[S16] J. Behler, "Atom-centered symmetry functions for constructing high-dimensional neural network potentials," J. Chem. Phys. **134**, 74106 (2011).

[S17] A. Singraber, T. Morawietz, J. Behler, and C. Dellago, "Parallel Multistream Training of High-Dimensional Neural Network Potentials," J. Chem. Theory Comput. **15**, 3075–3092 (2019).

[S18] W. L. Jorgensen, J. Chandrasekhar, J. D. Madura, R. W. Impey, and M. L. Klein, "Comparison of simple potential functions for simulating liquid water," J. Chem. Phys. **79**, 926–935 (1983).

[S19] V. Quaranta, M. Hellström, and J. Behler, "Proton-Transfer Mechanisms at the Water-ZnO Interface: The Role of Presolvation," J. Phys. Chem. Lett. **8**, 1476–1483 (2017).

[S20] S. Yue, M. C. Muniz, M. F. Andrade, L. Zhang, R. Car, and A. Z. Panagiotopoulos, "When do short-range atomistic machine-learning models fall short?" Journal of Chemical Physics **154**, 34111 (2021).

[S21] T. D. Kühne, M. Iannuzzi, M. Del Ben, V. V. Rybkin, P. Seewald, F. Stein, T. Laino, R. Z. Khaliullin, O. Schütt, F. Schiffmann, D. Golze, J. Wilhelm, S. Chulkov, M. H. Bani-Hashemian, V. Weber, U. Borštnik, M. Taillefumier, A. S. Jakobovits, A. Lazzaro, H. Pabst, T. Müller, R. Schade, M. Guidon, S. Andermatt, N. Holmberg, G. K. Schenter, A. Hehn, A. Bussy, F. Belleflamme, G. Tabacchi, A. Glöß, M. Lass, I. Bethune, C. J. Mundy, C. Plessl, M. Watkins, J. VandeVondele, M. Krack, and J. Hutter, "CP2K: An electronic structure and molecular dynamics software package - Quickstep: Efficient and accurate electronic structure calculations," J. Chem. Phys. **152**, 194103 (2020).

[S22] F. G. Fumi and M. P. Tosi, "Ionic sizes and born repulsive parameters in the NaCl-type alkali halides—I: The Huggins-Mayer and Pauling forms," J. Phys. Chem. Solids **25**, 31–43 (1964).

[S23] R. M. Lynden-Bell and J. C. Rasaiah, "From hydrophobic to hydrophilic behaviour: A simulation study of solvation entropy and free energy of simple solutes," J. Chem. Phys. **107**, 1981–1991 (1997).

[S24] A. L. Benavides, J. L. Aragones, and C. Vega, "Consensus on the solubility of NaCl in water from computer simulations using the chemical potential route," The Journal of Chemical Physics **144**, 124504 (2016).

[S25] S. Grimme, J. Antony, S. Ehrlich, and H. Krieg, "A consistent and accurate ab initio parametrization of density functional dispersion correction (DFT-D) for the 94 elements H-Pu," J. Chem. Phys. **132**, 154104 (2010).

[S26] S. Goedecker, M. Teter, and J. Hutter, "Separable dual-space Gaussian pseudopotentials," Phys. Rev. B **54**, 1703–1710 (1996).

[S27] J. VandeVondele and J. Hutter, "Gaussian basis sets for accurate calculations on molecular systems in gas and condensed phases," J. Chem. Phys. **127**, 114105 (2007).

[S28] G. Bussi, D. Donadio, and M. Parrinello, "Canonical sampling through velocity rescaling," J. Chem. Phys. **126**, 14101 (2007).

[S29] P. J. Steinhardt, D. R. Nelson, and M. Ronchetti, "Bond-orientational order in liquids and glasses," Phys. Rev. B **28**, 784–805 (1983).

[S30] W. Lechner and C. Dellago, "Accurate determination of crystal structures based on averaged local bond order parameters," J. Chem. Phys. **129**, 114707 (2008).

[S31] V. Ramasubramani, B. D. Dice, E. S. Harper, M. P. Spellings, J. A. Anderson, and S. C. Glotzer, "freud: A software suite for high throughput analysis of particle simulation data," Comput. Phys. Commun. **254**, 107275 (2020).

[S32] M. Bonomi, G. Bussi, C. Camilloni, G. A. Tribello, P. Banáš, A. Barducci, M. Bernetti, P. G. Bolhuis, S. Bottaro, D. Branduardi, R. Capelli, P. Carloni, M. Ceriotti, A. Cesari, H. Chen, W. Chen, F. Colizzi, S. De, M. De La Pierre, D. Donadio, V. Drobot, B. Ensing, A. L. Ferguson, M. Filizola, J. S. Fraser, H. Fu, P. Gasparotto, F. L. Gervasio, F. Giberti, A. Gil-Ley, T. Giorgino, G. T. Heller, G. M. Hocky, M. Iannuzzi, M. Invernizzi, K. E. Jelfs, A. Jussupow, E. Kirilin, A. Laio, V. Limongelli, K. Lindorff-Larsen, T. Löhr, F. Marinelli, L. Martin-Samos, M. Masetti, R. Meyer, A. Michaelides, C. Molteni, T. Morishita, M. Nava, C. Paissoni, E. Papaleo, M. Parrinello, J. Pfaendtner, P. Piaggi, G. M. Piccini, A. Pietropaolo, F. Pietrucci, S. Pipolo, D. Provasi, D. Quigley, P. Raiteri, S. Raniolo, J. Rydzewski, M. Salvalaglio, G. C. Sosso, V. Spiwok, J. Šponer, D. W. Swenson, P. Tiwary, O. Valsson, M. Vendruscolo, G. A. Voth, and A. White, "Promoting transparency and reproducibility in enhanced molecular simulations," Nat. Methods **16**, 670–673 (2019).

[S33] L.-M. Liu, A. Laio, and A. Michaelides, "Initial stages of salt crystal dissolution determined with ab initio molecular dynamics," Phys. Chem. Chem. Phys. **13**, 13162–13166 (2011).
